# A novel gene signature based on five immune checkpoint genes predicts the survival of glioma

**DOI:** 10.1186/s41016-020-00220-2

**Published:** 2021-02-03

**Authors:** Wei Zhang, You Zhai, Guanzhang Li, Tao Jiang

**Affiliations:** 1 Department of Neurosurgery, Beijing Tiantan Hospital, Capital Medical University, Beijing, China; 2 Department of Molecular Neuropathology, Beijing Neurosurgical Institute, Capital Medical University, No. 119 South Fourth Ring Road West, Fengtai District, Beijing, China; 3 Center of Brain Tumor, Beijing Institute for Brain Disorders, Beijing, China; 4 China National Clinical Research Center for Neurological Diseases, Beijing, China; 5 Chinese Glioma Genome Atlas Network (CGGA) and Asian Glioma Genome Atlas Network (AGGA), Beijing, China

**Keywords:** Glioma, Immune checkpoints, Gene signature, Prognosis

## Abstract

**Background:**

Glioma is the most common and fatal type of nerve neoplasm in the central nervous system. Several biomarkers have been considered for prognosis prediction, which is not accurate enough. We aimed to carry out a gene signature related to the expression of immune checkpoints which was enough for its performance in prediction.

**Methods:**

Gene expression of immune checkpoints in TGGA database was filtrated. The 5 selected genes underwent verification by COX and Lasso-COX regression. Next, the selected genes were included to build a novel signature for further analysis.

**Results:**

Patients were sub-grouped into high and low risk according to the novel signature. Immune response, clinicopathologic characters, and survival showed significant differences between those 2 groups. Terms including “naive,” “effector,” and “IL-4” were screened out by GSEA. The results showed strong relevance between the signature and immune response.

**Conclusions:**

We constructed a gene signature with 5 immune checkpoints. The signature predicted survival effectively. The novel signature performed more functional than previous biomarkers.

**Supplementary Information:**

The online version contains supplementary material available at 10.1186/s41016-020-00220-2.

## Background

Glioblastoma (GBM) is the most common and lethal type of intracranial malignancy [1]. But so far, the effect of modern comprehensive medical care for those lethal conditions is limited. The median survival is only about 14.4 months [2]. Several prediction models based on RNA sequences were produced to anticipate the survival and prognosis of patients, yet the outcomes cannot reach the ideal accuracy [3].

For the past few years, molecular pathology characterized by IDH status and methylation of O-methylguanine DNA methyltransferase (MGMT) showed greater accuracy in predicting therapeutic effects and prognosis [3, 4]. With the help of genome databases, we can filtrate several biomarkers between normal tissue and tumor, which drives us into a new era in data analysis. However, the accuracy of the anticipation based on a single gene or signature was not effective enough [5-7]. Multi-gene combination analysis is a possibly better alternative.

Immune checkpoints are the regulator of the immune system. Low immune activity in glioblastoma largely due to the unbalance between stimulatory and inhibitory checkpoints and certainly produces poor prognosis [8, 9]. Suggesting the importance of introducing immune checkpoints into the evaluation of prognosis.

In this article, a signature based on the expression of several immune checkpoints was to predict the survival and prognosis of GBM patients. First, we identified corresponding immune checkpoint genes, which are highly correlated with prognosis using COX text based on TCGA and CGGA. Then we launched Lasso-COX analysis based on genes identified previously. Finally, we enrolled five genes, which are associated with overall survival, for the novel rick score. Patients with higher signatures have a poorer prognosis than those with a lower score. The conclusion can be dual authenticated in both TCGA and CGGA.

## Methods

### Patient cases and data processing

In all, 631 patients of TCGA were involved as an experimental group, while 325 patients of CGGA database were included as an independent verification group. Each case from CGGA was diagnosed and followed-up confirmedly. Tumor samples were acquired from newly removed tissue. All cases from CGGA underwent treatment by members of the CGGA group. RNA sequencing was only proceeded when tumor cells accounting for more than 80% of the total volume of the tumor bulks. Overall survival (OS) was determined from the date of diagnosis to the end of follow-up, including death or the latest follow-up. The date of death was determined by the certification from police stations. Cases information from TCGA was downloaded from TCGA official website (http://cancergenome.nih.gov/).

### Gene selection and signature building

COX analysis was performed on the TCGA data. Sixteen immune checkpoints statistically correlated with prognosis were selected. Five in the above 16 immune checkpoints were further filtered using lasso-COX dimension reduction analysis, which was B7-H6, CD40, OX40 Ligand, PD-L1, and TIM-3. These genes were highly associated with prognosis (*P* < 0.05) in TCGA. The hazard ratio of each of the five genes was enrolled to construct the gene signature. The signature was independently verified in CGGA.

$$ signature\ risk\ score=\sum \limits_{i=1}^n{\beta}_i{x}_i $$

Said *β*_*i*_ indicates the hazard rate for each gene in TCGA, *x*_*i*_ stands for the gene expression value of each gene. The signature, formed by 5 genes, was determined by a linear combination of the expression weighted with regression coefficients from Lasso-Cox regression model.

### Statistical analysis

Patients from TCGA and CGGA were sub-grouped into a high and low risk based on their median signature. The heatmap was produced to evaluate the relationship between clinical characteristic and the signature. The prognostic significance was assessed by the method of Kaplan-Meier curves. To analyze the accuracy of prediction, we used the ROC curve. GSEA analysis was carried out to reflect different DNA expression between high-risk and low-risk groups. All statistical analyses were conducted using R (https://www.r-project.org/, v3.4.1), SPSS 16.0 (SPSS Inc., Chicago, IL), and GraphPad Prism 7 (GraphPad Software Inc., La Jolla, CA). GSEA and analysis were implemented with the java software GSEA (http://software.broadinstitute.org/gsea/index.jsp)

## Results

### Identification of the included genes

**Fig. 1:**
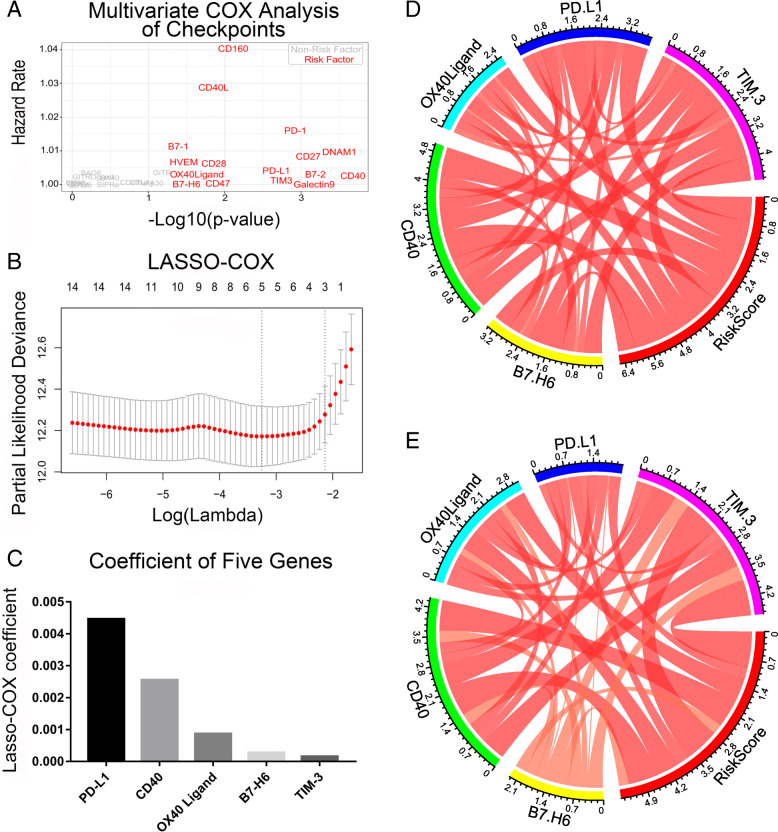
Five immune checkpoints related genes were identified. **a** Filtrating target genes with multivariate COX analysis. Independent correlated immune checkpoints were shown in red. **b** Five genes were selected by lasso-COX. **c** Coefficient of the 5 genes. The coefficients of each immune checkpoint were shown in the figure. **d** and **e** Relationship between the 5 genes and signature

We first collected all glioma-associated immune checkpoints by reviewing the literature and analyzed each of the immune checkpoints independently using multivariate COX analysis based on the expression level in TCGA. Sixteen immune checkpoints that were statistically relevant with prognosis independently were selected (Fig. 1a). Taking a further step, we performed Lasso-COX dimensionality reduction analysis on the 16 mentioned immune checkpoints based on their corresponding expression level displayed in the TCGA database. Five marked correlation genes in TCGA database were selected (Fig. 1b), including PD-L1, CD40, OX40 Ligand, B7-H6, and TIM3. The dependency was shown in Fig. 1c. The expression level of all selected genes is positively correlated with the signature. They were involved to construct the predictive signature. The circle maps referred to the strong relationship among the 5 immune checkpoints and signatures in both TCGA and CGGA (Fig. 1d and e). All checkpoints involved in signature construction showed a slight difference (B7-H6) or no difference (the other four) in primary and recurrent glioma samples from TCGA (Fig. S1).

### The signature was connected with clinicopathologic characteristics but performed better in prognosis prediction

**Fig. 2:**
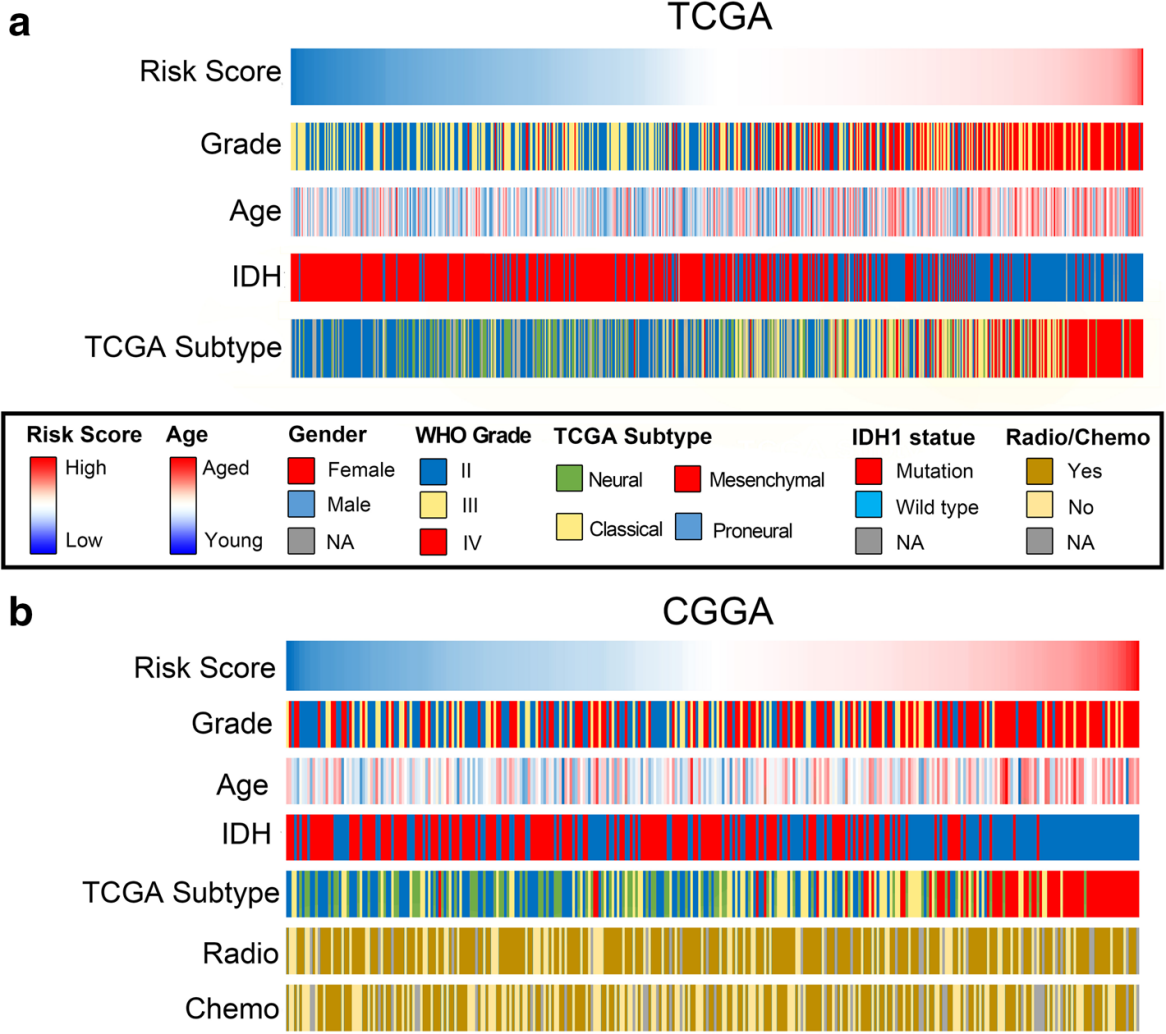
Connection of the signature and the common clinical characteristics. **a** The clinicopathologic information of patients in TCGA, data was arranged by the increasing signature. **b** The clinicopathologic information of patients in CGGA database, arranged by the increasing signature

The signature of 631 patients of TCGA and 325 patients of CGGA were calculated respectively. We further investigated if the signature was correlated with clinicopathologic characteristics. Patients were ranked according to their signature, and both TCGA and CGGA showed a similar distribution in regard to the IDH status, grade, age, and TCGA subtype as exhibited in Fig. 2. To sum up, younger aged, IDH mutant, low grade, and non-mesenchymal subtype patients were skewed to the lower risk group. Elder aged, IDH wild type, high grade, and mesenchymal subtype patients were mainly enriched in the higher risk section. However, chemo- or radiotherapy intervention was not related to signature, indicating it was a comprehensive expression of immune checkpoints that influence prognosis, rather than treatment.

**Fig. 3:**
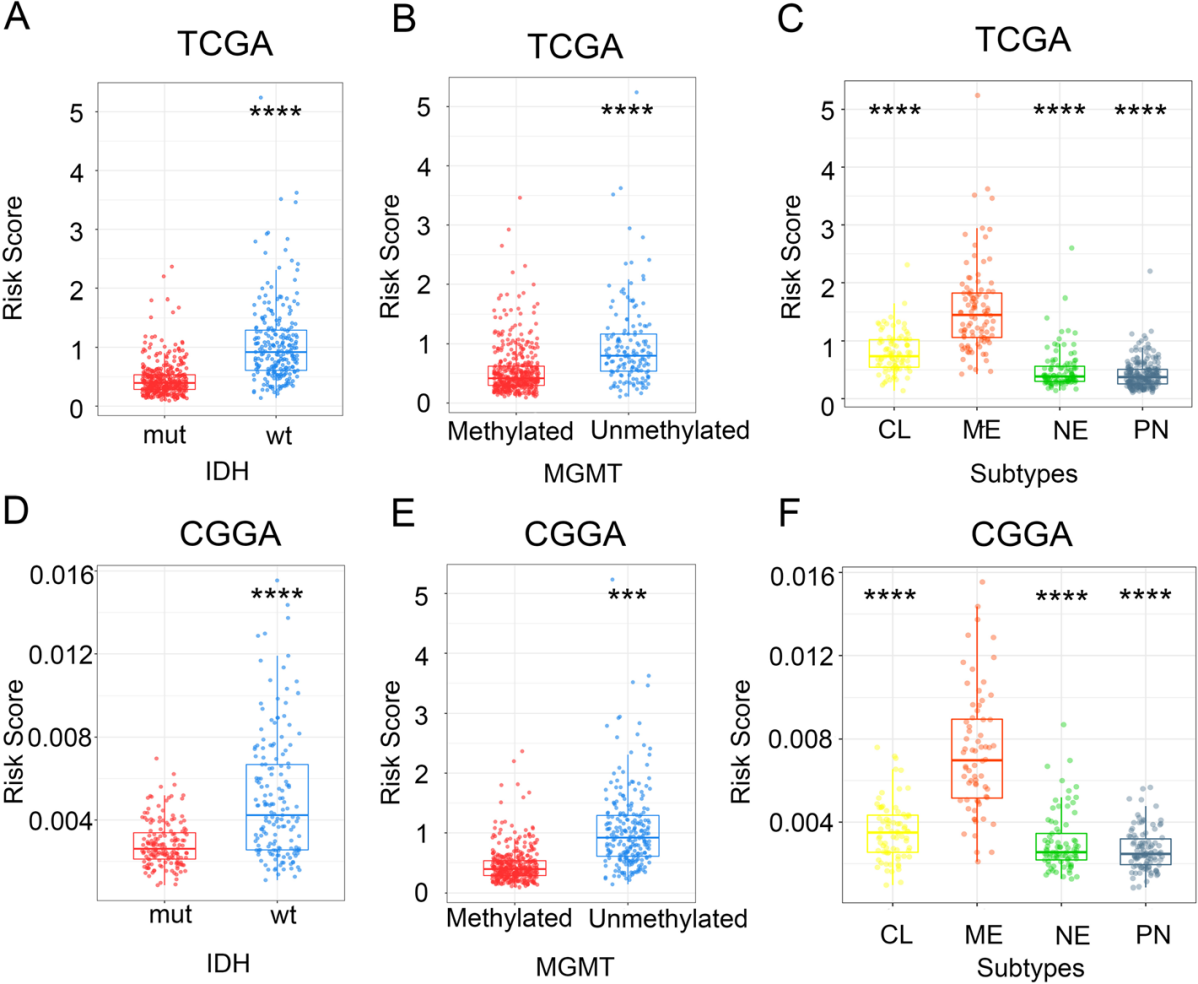
The signature and common clinicopathologic characteristics. **a**-**c** Distribution of the signature in patients of TCGA stratified by IDH status (**a**), MGMT promoter methylation (**b**), and TCGA subtypes (**c**). ***P < 0.001, ****P < 0.0001. **d**-**f** Distribution of the signature in patients of CGGA stratified by IDH status (**d**), MGMT promoter methylation (**e**), and TCGA subtypes (**f**). ***P < 0.001, ****P < 0.0001

We further compared the distribution of the signature based on the stratification of the subtypes of molecular and pathology characteristics. The signature was significantly different between groups layered by IDH mutant status, MGMT promoter methylation, and the TCGA subtypes (Fig. 3). The signature soared in the IDH wild-type sub-group in TCGA. Similar phenomenon was repeated in CGGA (Fig. 3a and d). A similar situation was observed in the analysis in line with MGMT promoter methylated, as well as the mesenchymal subtype patients (Fig. 3b, c, e, f). The results indicated that the comprehensive immune status was, to some extent, correlated with molecular and pathology characteristics.

To compare the effectiveness of the signature and other clinicopathologic characteristics, we performed uni- and multivariate COX analysis. According to the result of the univariate COX regression, IDH status, MGMT promoter status, grade, age, and the signature were all significantly correlated to the survival (Table 1). Yet the signature was considered to be the most significant factor correlated with the survival in CGGA (HR > 10, *P* = 0.025). The results above referred the signature performed better than other clinicopathologic characteristics. Similar results were reproduced in TCGA database by the similar methods.

**Table 1:** Uni- and multivariate Cox regression analysis of the risk score and clinicopathologic factors for OS in TCGA and CGGA databases

Variable	Univariate Cox			Multivariate Cox		
	*P* value	HR	95% CI for HR	*P* value	HR	95% CI for HR
Gender	0.350	1.145	0.862-1.519			
Age	<0.0001	1.073	1.061-1.084	<0.0001	1.057	1.043-1.072
IDH 1 status	<0.0001	0.163	0.121-0.219	0.085	0.726	0.504-1.045
MGMT promoter status	<0.0001	0.337	0.247-0.461	0.067	0.712	0.494-1.025
Grade	<0.0001	4.794	3.798-6.051	<0.0001	2.071	1.538-2.788
RiskScore	<0.0001	3.182	2.682-3.777	<0.0001	2.050	1.620-2.593
Variable	Univariate Cox			Multivariate Cox		
	*P* value	HR	95% CI for HR	*P* value	HR	95% CI for HR
Gender	0.345	1.181	0.837-1.666			
Age	<0.0001	1.038	1.023-1.054	0.368	1.007	0.991-1.023
IDH 1 status	<0.0001	0.256	0.178-0.368	0.165	0.687	0.405-1.167
MGMT promoter status	<0.0001	0.516	0.362-0.736	0.077	0.706	0.480-1.039
Grade	<0.0001	3.477	2.716-4.452	<0.0001	2.513	1.850-3.415
Chemotherapy	0.125	1.151	0.962-1.378			
Radiotherapy	0.688	0.945	0.718-1.244			
RiskScore	<0.0001	>10	>10	0.025	>10	>10

### Gene expression and signature

**Fig. 4:**
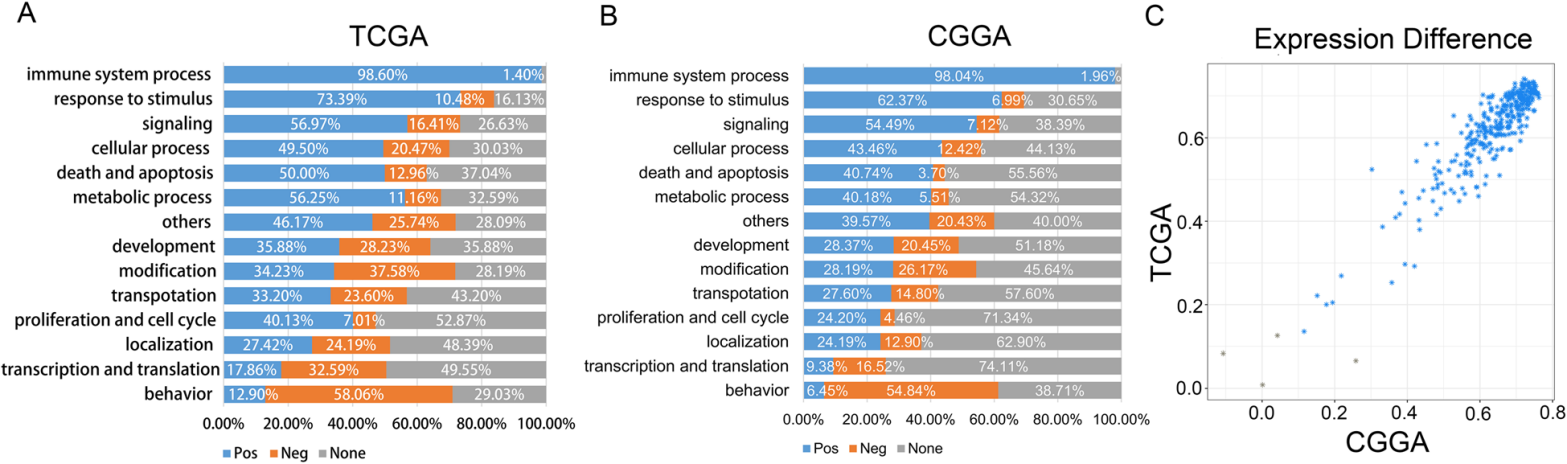
The signature and gene function enrichment. **a**-**b** GO terms where the positively enriched in the label of “immune system process.” **c** The expression difference of genes related to the immune system process between samples in TCGA and CGGA

We calculated the relationship between the signature and different cellular functions (Fig. 4a and b). As shown in the result, the signature showed a distinctly positive relationship with the immune system process in both TCGA and CGGA. Over 98% of gene expression correlated with the immune system process behaved a positive relationship with the signature. Meanwhile, all mentioned genes for the immune system process distributed similarly both in TCGA and CGGA (Fig. 4c). Besides, the portion of signature positive-related gene expression for response to stimulus and signaling is relatively higher than other function sections (more than 60% and 50% respectively). Intriguingly, the function of behavior showed a remarkable negative relationship with the signature in both databases, indicating the immunosuppression effect in the tumor micro-environment.

**Fig. 5:**
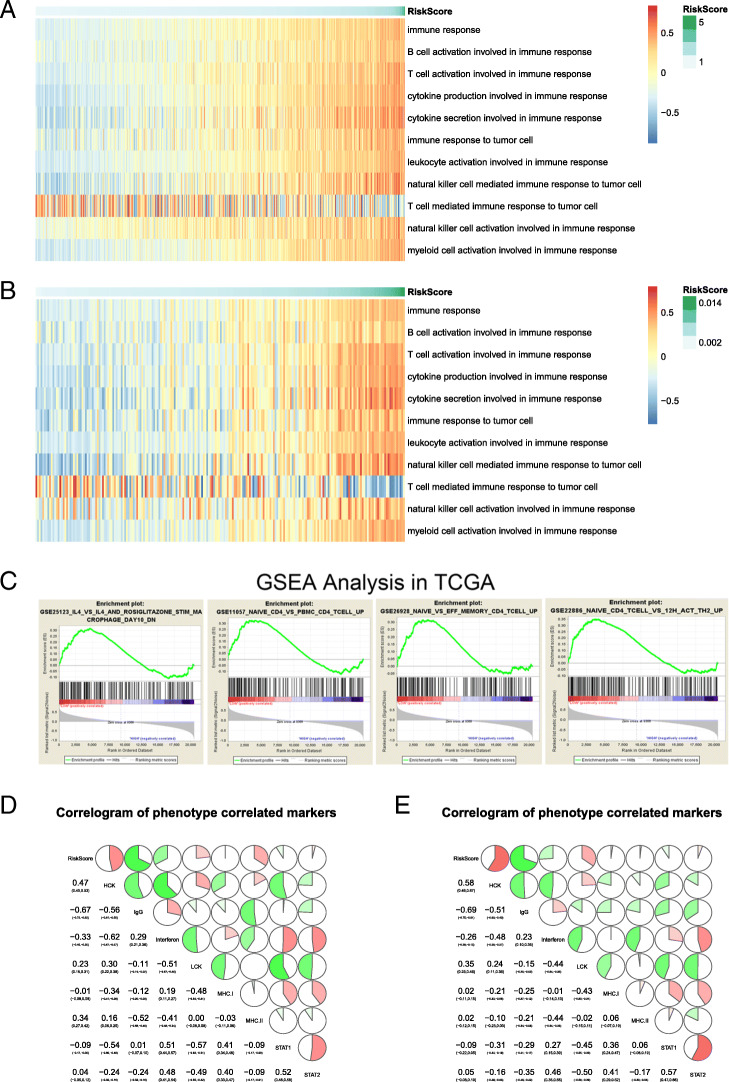
Stratification of genes for the immune system process. **a**-**b** The immunological related gene expression information of patients in TCGA and CGGA database, arranged by the increasing signature. **c** Hallmarks enriched in the high-signature group. **d** Correlation between inflammatory factors and signature

Taking a further step, we subdivided the gene list of “immune system process” mentioned in Fig. 4. The gene list was separated into several sections as shown in Fig. 5a and b. The result showed that most of immune-associated gene expression was positively related to signature in TCGA database (Fig. 5a). But in contrast, “T cell mediated immune response to tumor cell” was negatively related to the signature, indicating there were plenty of lymphocytes involved in the tumor micro-environment but most of them were “bystanders.” The phenomenon implied that the upregulation of the immune checkpoints involved in the construction of the signature attribute to the T cell exhaustion during immune response in tumor, and further cause poorer prognosis (Fig. 5a). The result was similar in CGGA database (Fig. 5b).

Meanwhile, differences in gene expression among the high and low score of the signature groups in TCGA were enriched by Gene Set Enrichment Analysis (GSEA). We noticed that hallmarks including “IL-4” and “naive” were significantly enriched in the high-signature group in TCGA (Fig. 5c). The same trend was repeated in the same analysis in CGGA (Fig. S2). Whereas, terms including “effector” were significantly enriched in the low-risk group. Besides, inflammatory-related biomarkers, including interferon and its downstream signal STAT1, showed a negative correlation with the signature (Fig. 5d and e). On the contrary, proteins related to T cell activation, such as LCK, presented a positive correlation with the signature (Fig. 5d), implying the dysfunction of T cells happened in glioma. All mentioned results suggest that the novel signature reflects the immunosuppressive effect related to cytokine and effector lymphocyte portion in the tumor microenvironment.

### The signature provided ideal prediction effectiveness both in TCGA and CGGA

**Fig. 6:**
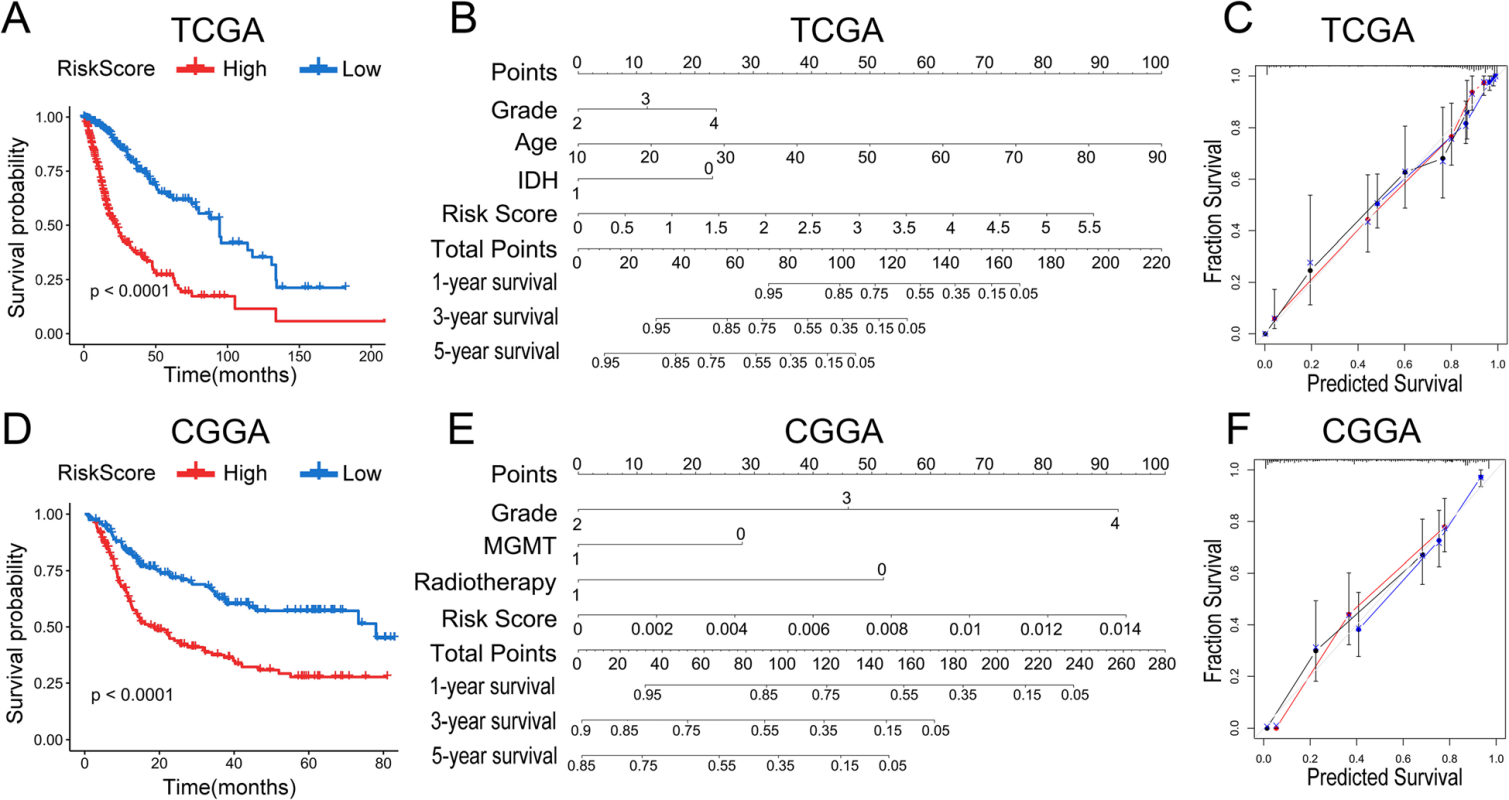
Clinical accuracy of the signature. **a** and **c** The overall survival of high- and low-signature group in TCGA and CGGA database, respectively. **b** and **e** Nomogram rating scale was constructed with clinicopathological characteristics and the signature according to TCGA and CGGA, respectively. **c** and **f** Predictive accuracy assessment of TCGA and CGGA, respectively

High- and low-risk groups in TCGA and CGGA were divided based on the median score of the signature (cut-off) of all patients, respectively. We evaluated the survival period of each group and found it was remarkably shorter in the high-risk group than the low-risk group (*P* < 0.0001) (Fig. 6a and d). Then we built a nomogram rating scale for survival period prediction (Fig. 6b and e). As the scale shown in Fig. 6b, which was built based on TCGA, different index (grade, age, IDH status, signature) corresponds to the particular points displayed in the first line respectively. The corresponding values were added together to become the total score, which was used to estimate the probability of survival in different periods. Some indexes were altered in the CGGA nanogram scale as shown in Fig. 6e. In all, patients with higher grade, elder aged, IDH wild type, MGMT promoter unmethylated, and higher score of the signature are tended to have a poorer prognosis. The accuracy of the prediction of both scales was evaluated and was depicted in Fig. 6c and f, corresponding to TCGA and CGGA respectively.

## Discussion

Numerous works confirmed the extreme heterogeneity of GBM, which makes the prognosis and therapy of primary GBM difficult [10]. Recently, classical clinicopathological characteristics, such as age, gender, IDH status, and MGMT promoter status, are not sufficient to predict the prognosis accurately neither in univariate nor in multivariate methods. Therefore, gene-based biomarker filtration becomes a hotspot [11], but single-gene prediction models are still not specific enough. Generally, a single gene can be influenced by multiple factors. In the meantime, based on our years of clinical experience, the detection of a single immune checkpoint in a single patient (susceptible to sampling and other factors) is highly unstable. These objective circumstances make the method of single-gene prediction model difficult to be popularized in clinical practice. Immunotherapy has been a hot topic in recent years. The immune checkpoint is the key factor affecting immunotherapy. Recently, studies of individual targets (PD1/TIM3/PDL1, etc.) have been reported. But there is no comprehensive analysis that has been published. We constructed a gene signature enrolling all known immune checkpoints, with the purpose of not only predicting prognosis but also guiding multi-immune checkpoint blocked therapy and clinical detection kit development. This model is widely accepted and superior to the single gene prediction model in the prognosis of diseases [12]. Although those included genes showed inferior predictive accuracy in prognosis when have been involved for prediction alone, this model can cover the shortage and promote predictive effect. Similar works had been launched previously [13].

Given the activity of T cells mainly attributes to the expression of immune checkpoints and the necessity of T cells in tumor immunity [14], it is reasonable to investigate the contribution of gene expression of immune checkpoints in GBM patients. Therefore, in this article, we extracted all known immunosuppressive immune checkpoints in both TCGA and CGGA for signature constructing.

The five selected genes involved to construct that the signature were functionally independent from each other [15]. The performance of the novel signature was assessed in TCGA and CGGA, separately. Patients in the high-risk group have significantly shorter survival than those in the low-risk group. Besides, the signature was proved to be an independent and robust prognostic factor which surpassed all the other common clinical parameters.

The signature was highly related but independent to classic molecular characteristics such as IDH status, MGMT promoter methylation, and TCGA subtype, which is consistent with some previous work by us and others [16-18]. The univariate and multivariate Cox result confirmed the signature to function as an independent prognostic factor with higher predictive accuracy than other clinicopathologic characteristics.

As expected, the signature was remarkably related to biomarkers of immune response. GO analysis was further launched; the top six gene function most relevant to the signature was all about the immune response (Fig. S3). Intriguingly, the activation of the immune system was positively related to the signature. Most immune response-related gene expression and part of T cell activation associated proteins (such as Lck) were positively related to the signature. On the contrary, “naive” and IL-4 (immunosuppressive cytokine) were enriched in the high-risk group. Interferon and “T cell-mediated immune response to tumor cell” was also negatively related to the signature. Intriguingly, in all subtypes of interferon, only interferon *γ* was correlated, and negatively related to the signature of patients from both database (Fig. S4). Such a contradiction indicated remarkable cell recruitment and activation happened in the tumor micro-environment, at least at the beginning of tumorigenesis. But these measures are futile since cells that were recruited and activated were not able to function as it should. Although these cells may retain the ability to kill tumors, their normal function was inhibited due to upregulated expression at some immune checkpoints. This also indirectly proved the necessity of comprehensive immune checkpoint antagonism therapy.

## Conclusions

In summary, we built a novel gene signature based on 5 immune checkpoints selected from all known immune checkpoints. The novel signature produced a promising method for prognosis prediction. This model can also serve as a tool for patient stratification during clinical word. The poor prognosis for patients in high-risk provided new evidence for the relationship between malignancy and immune checkpoints. Finally, the novel nomogram rating scale was built for higher accuracy and reliability prognosis prediction. Although the clinical detection kit based on the signature is still developing, several studies have revealed great influence and predictive value of immune checkpoints in oncology. Our model can be an ideal tool for improving the predictability of the prognosis. Thus, the prediction model based on the 5 immune checkpoints in GBM has the potential to guide individual multi-immune checkpoint blocked treatment.

## Supplementary Information

**Additional file 1: Fig. S1.** Difference in the expression level of single immune checkpoint involved in model constructing. In all, no significant difference was found between primary and recurrent glioma, except B7-H6. In the meantime, no difference in signature was observed in the two groups. The above results indicate that the prediction model is appropriate for both primary and recurrent patients.

**Additional file 2: Fig. S2.** GSEA analysis in CGGA. The trend showed in Fig 5. C was repeated in CGGA database. Similarly, “Naive” and “IL-4” were enriched in high risk group. The above results indicate that immunosuppression happened in high risk group.

**Additional file 3: Fig. S3.** GO analysis in TCGA and CGGA database. The top 6 gene functions that were most correlated with signatures were listed. All functions are in respect to immune response. The result indicated that the upregulation of the checkpoints involved in model constructing triggered poor prognosis only by modulating the immune response.

**Additional file 4: Fig. S4.** The correlation of all subtypes of interferon and signature. Among all subtypes of interferon, only interferon γ was statistically relevant with the corresponding signature of patients both in TCGA and CGGA, simultaneously. Such a negative correlation indicated the function-loss of T cells post activation.

## Data Availability

The sequencing data, clinical, and follow-up information of primary and recurrent LGG patients were uploaded to the CGGA portal (http://cgga.org.cn/). All datasets used and/or analyzed in this study are available from the corresponding author on reasonable request.
