# Supplementary figures and images for: A novel gene signature based on five immune checkpoint genes predicts the survival of glioma
Source: Chin Neurosurg J. 2021 Feb 3;7:15. doi: 10.1186/s41016-020-00220-2 (PMC7856730; doi:10.1186/s41016-020-00220-2)

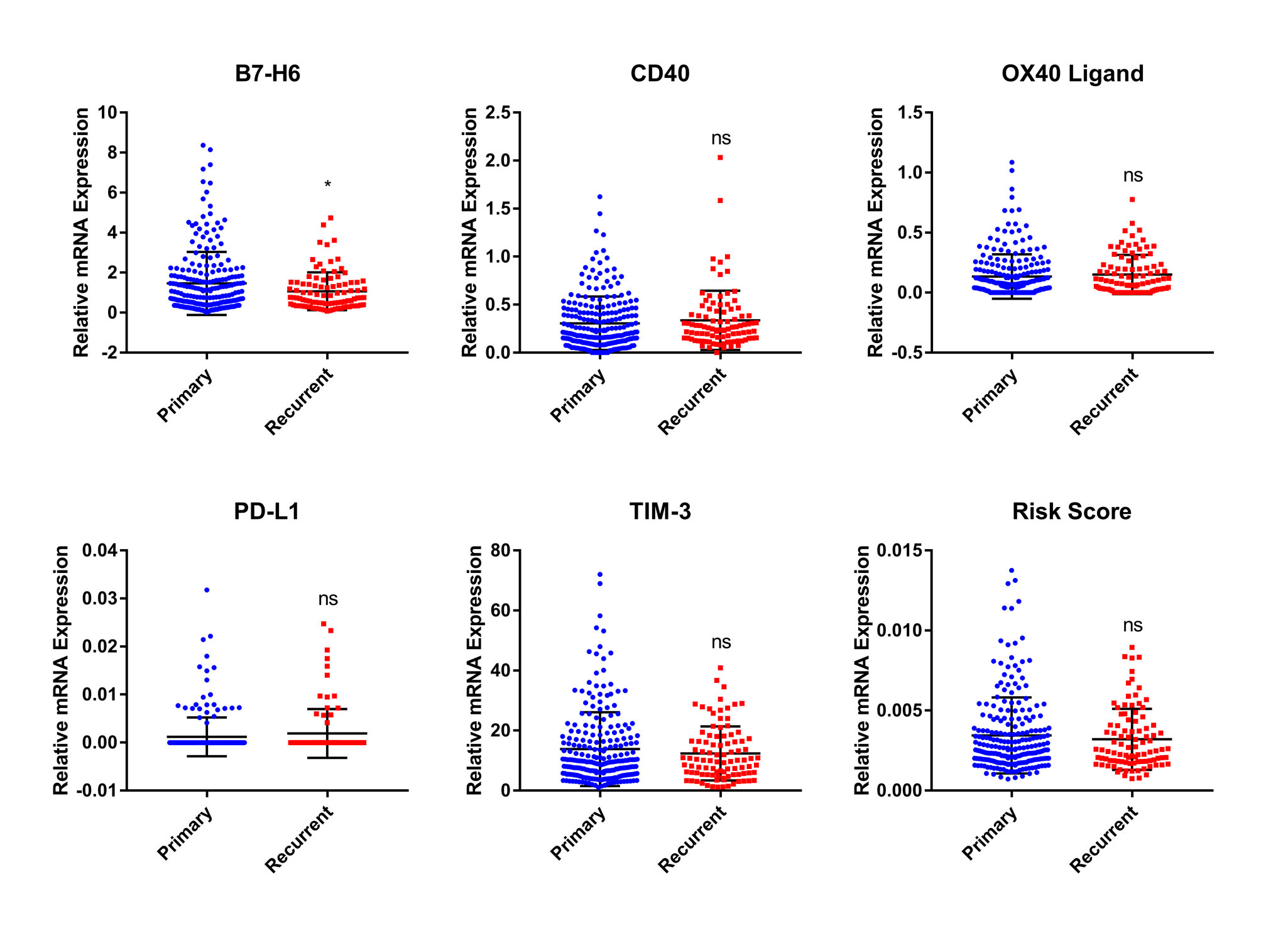

Supplement: Supplementary file 1 — Additional file 1: Fig. S1. Difference in the expression level of single immune checkpoint involved in model constructing. In all, no significant difference was found between primary and recurrent glioma, except B7-H6. In the meantime, no difference in signature was observed in the two groups. The above results indicate that the prediction model is appropriate for both primary and recurrent patients. [file 41016_2020_220_MOESM1_ESM.jpg]

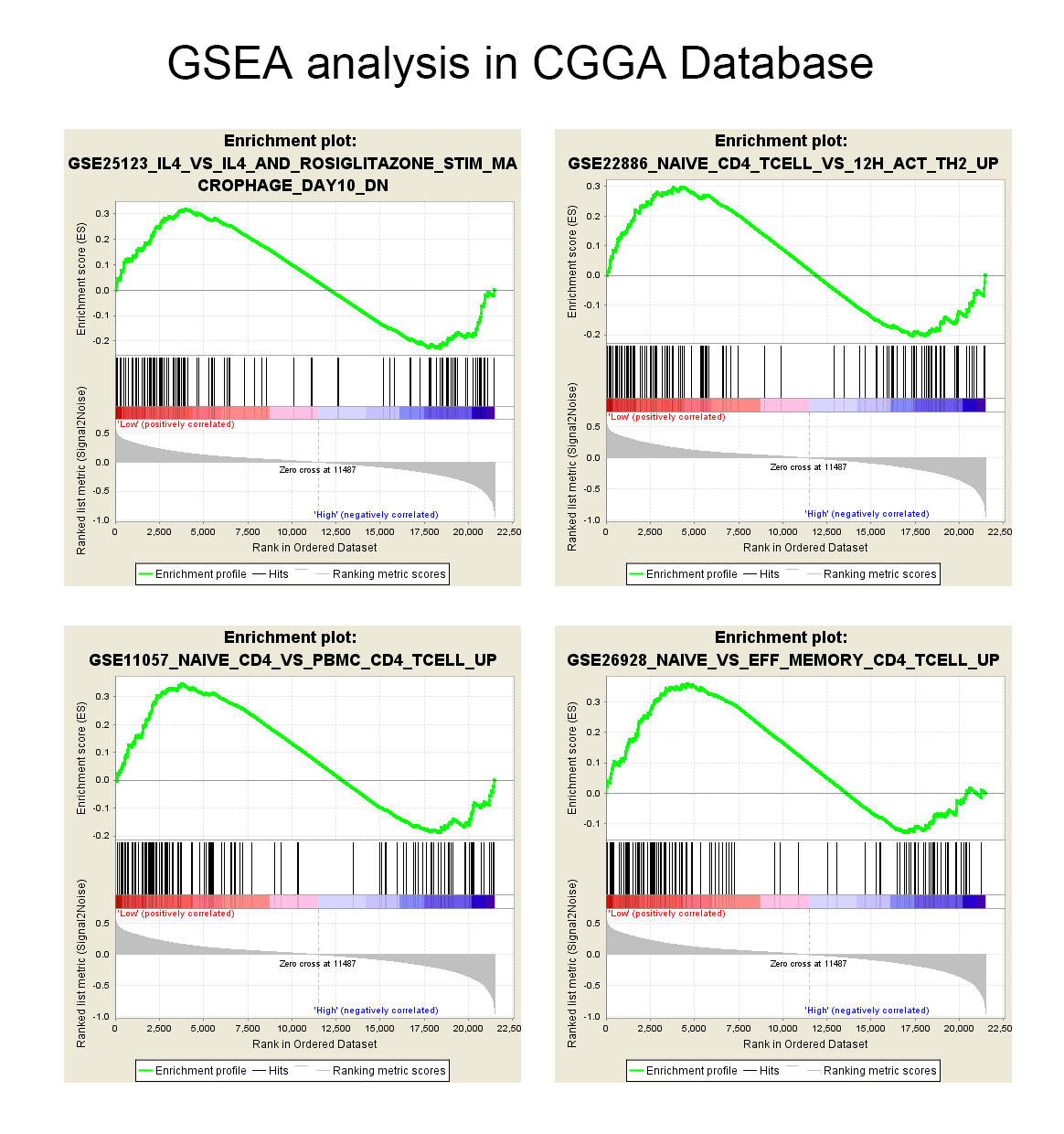

Supplement: Supplementary file 2 — Additional file 2: Fig. S2. GSEA analysis in CGGA. The trend showed in Fig 5. C was repeated in CGGA database. Similarly, “Naive” and “IL-4” were enriched in high risk group. The above results indicate that immunosuppression happened in high risk group. [file 41016_2020_220_MOESM2_ESM.jpg]

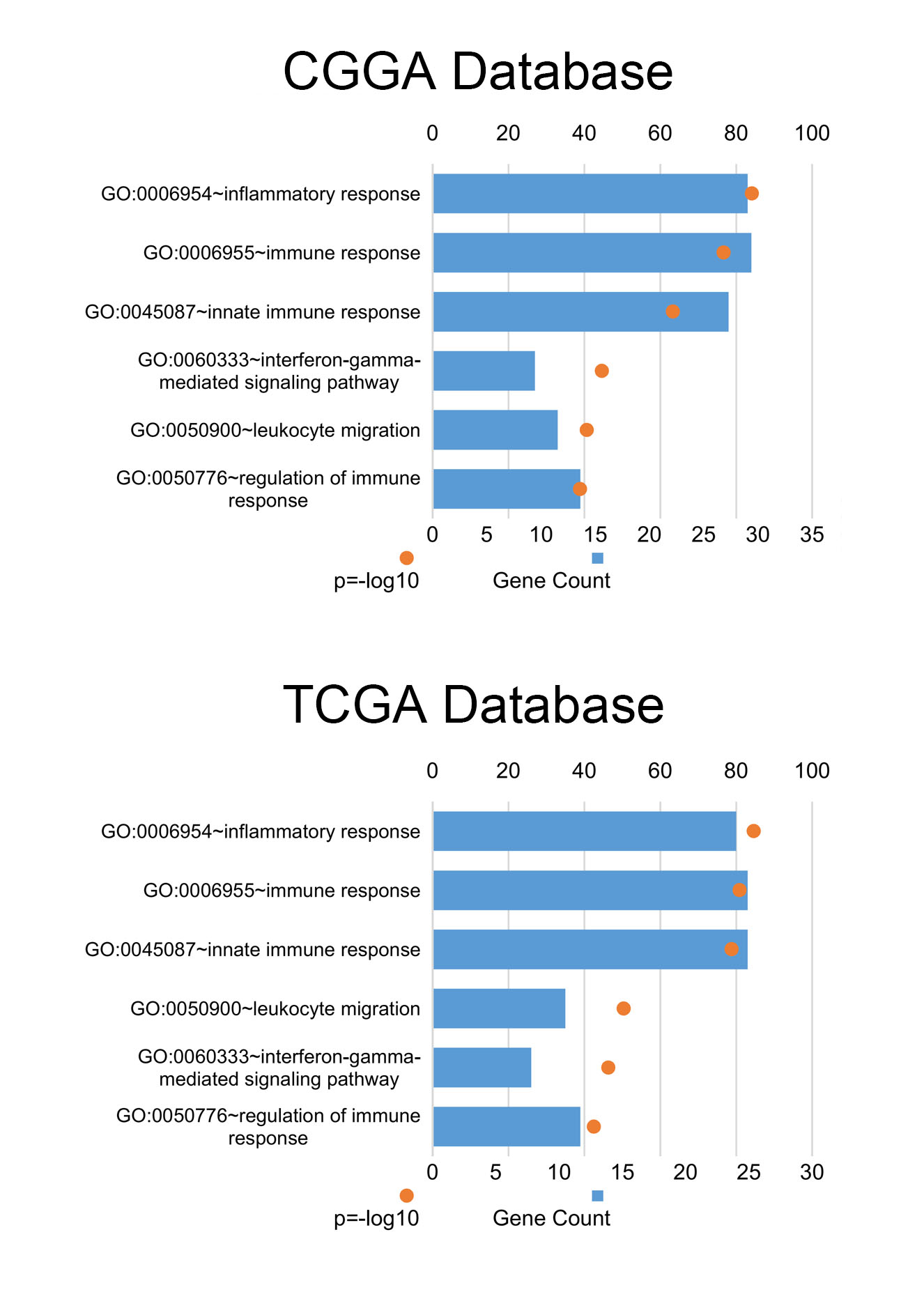

Supplement: Supplementary file 3 — Additional file 3: Fig. S3. GO analysis in TCGA and CGGA database. The top 6 gene functions that were most correlated with signatures were listed. All functions are in respect to immune response. The result indicated that the upregulation of the checkpoints involved in model constructing triggered poor prognosis only by modulating the immune response. [file 41016_2020_220_MOESM3_ESM.jpg]

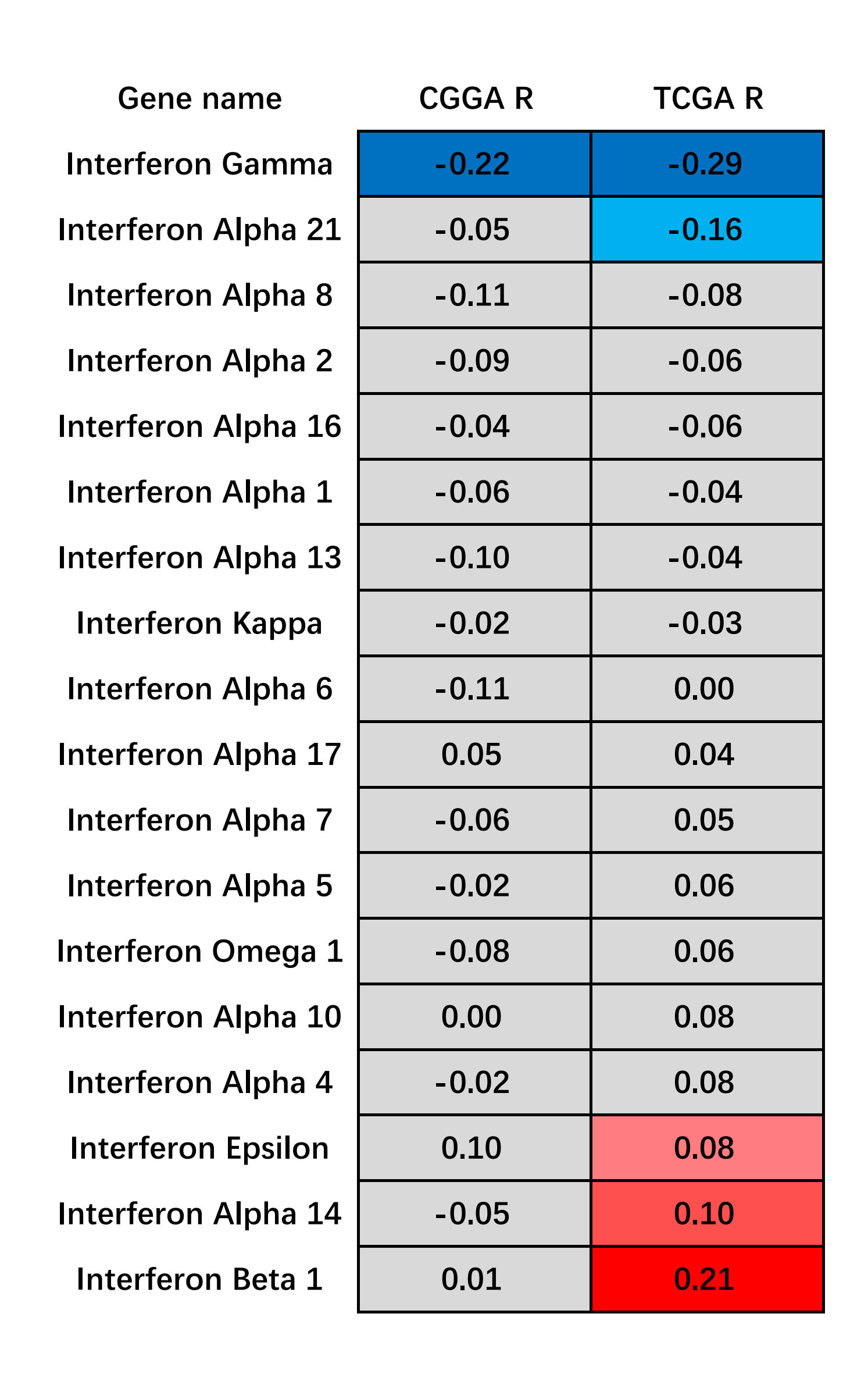

Supplement: Supplementary file 4 — Additional file 4: Fig. S4. The correlation of all subtypes of interferon and signature. Among all subtypes of interferon, only interferon γ was statistically relevant with the corresponding signature of patients both in TCGA and CGGA, simultaneously. Such a negative correlation indicated the function-loss of T cells post activation. [file 41016_2020_220_MOESM4_ESM.jpg]
